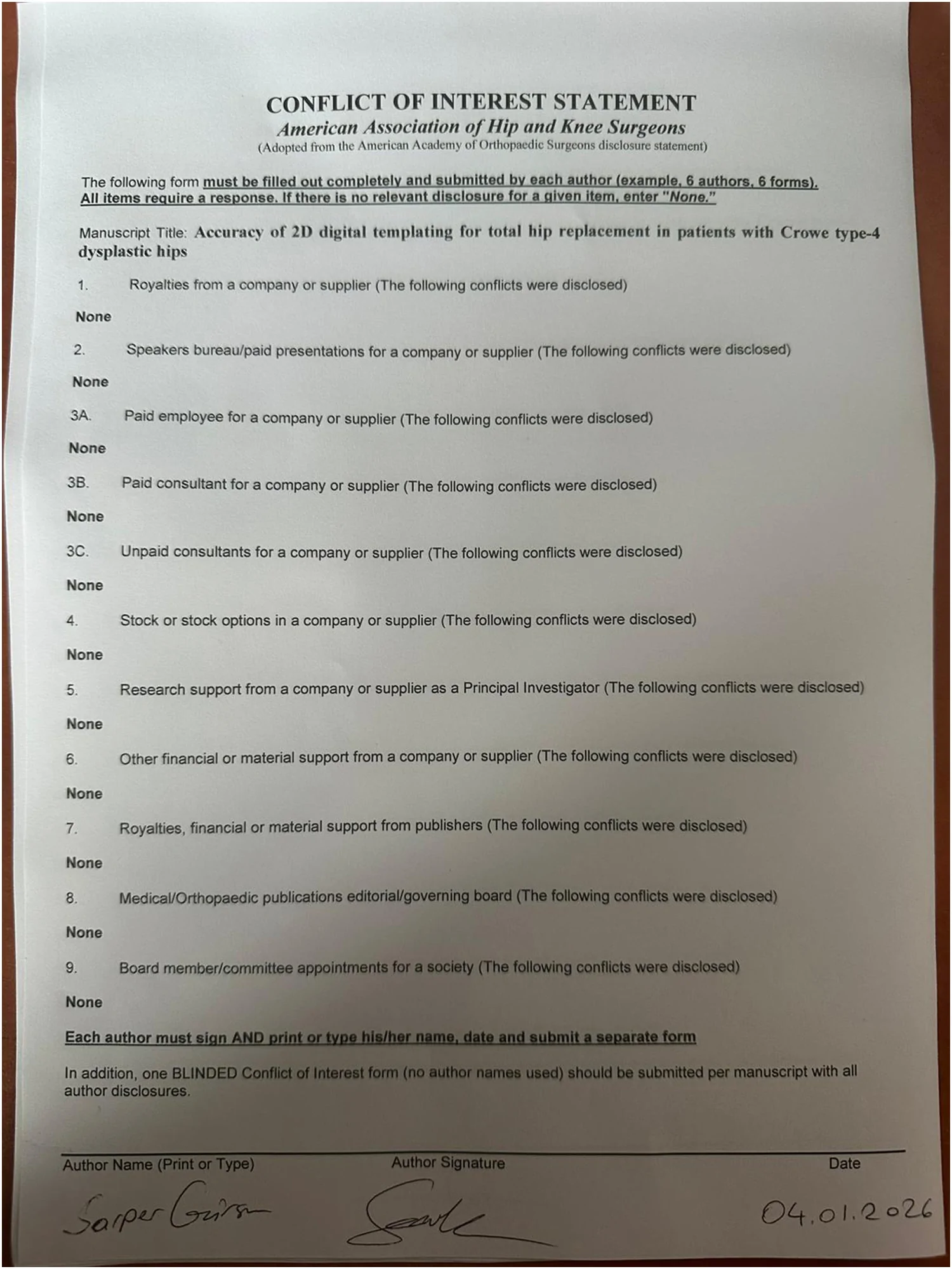# Accuracy of 2D Digital Templating for Total Hip Arthroplasty in Patients With Crowe Type-4 Dysplastic Hips

**DOI:** 10.1016/j.artd.2026.102182

**Published:** 2026-10-18

**Authors:** Ahmet Danacı, Ferdi Dırvar, Arman Vahabi, Atakan Ezici, Yakup Alpay, Sarper Gürsu

**Affiliations:** a Department of Orthopaedics and Traumatology, Medical Park Maltepe Hospital, İstanbul, Turkey; b Department of Orthopaedics and Traumatology, Baltalimani Bone and Joint Diseases Training and Research Hospital, İstanbul, Turkey; c Department of Orthopaedics and Traumatology, Ege University School of Medicine, İzmir, Turkey

**Keywords:** Developmental hip dysplasia, Preoperative planning, Femoral shortening, Leg length discrepancy

## Abstract

**Background:**

There is a paucity of studies reporting the outcomes of preoperative digital templating in Crowe type 4 hips. The aim of this study was to evaluate the accuracy and clinical role of digital templating applied in Crowe type 4 hips.

**Methods:**

Patient data were obtained from electronic patient records covering the period from January 2016 to December 2019. Data on side, gender, and age, the amount of femoral shortening, and the size of the implants were noted. Upon two dimensional (2D) digital templating, femoral implant size and positioning, length of femoral shortening, and acetabular implant size and positioning were predicted. The agreement for acetabular size, femoral size, and femoral shortening measurements was tested both for the senior arthroplasty surgeon and the senior resident.

**Results:**

Senior surgeon templating demonstrated moderate agreement with implanted acetabular (κ = 0.410) and femoral (κ = 0.432) component sizes, outperforming the senior resident (acetabular κ = 0.071; femoral κ = 0.265). Exact acetabular and femoral size prediction by the senior surgeon was 74% and 36%, respectively. Femoral shortening agreement was moderate for the surgeon (intraclass correlation coefficient [ICC] = 0.544) and fair for the resident (ICC = 0.400), whereas interobserver agreement for shortening was excellent (ICC = 0.958).

**Conclusions:**

The accuracy of 2D digital templating for predicting component size in total hip arthroplasty for Crowe type IV dysplastic hips was limited. Nevertheless, when performed by an experienced surgeon, 2D digital templating remains a valuable preoperative planning tool for planning and estimating the required femoral shortening, while final implant selection should be based on intraoperative findings.

## Introduction

Developmental hip dysplasia encompasses a spectrum of clinical scenarios, ranging from subtle dysplastic changes in the hip joint to complete dislocation affecting both acetabulum and proximal femur. Although its incidence has gradually decreased in recent years due to effective screening programs, various stages and clinical presentations of this condition are still observed in certain settings. While in most cases early diagnosis and simple interventions can yield satisfactory results in early detected cases, more complicated interventions such as total hip arthroplasty (THA) may be necessary in late, symptomatic cases [[1], [2], [3]].

Preoperative templating is a critical consideration in arthroplasty procedures. While it is not universally performed for every setting, many arthroplasty centers routinely employ templating in the preoperative assessment phase for hip and knee arthroplasties. Templating could provide valuable guidance to surgeons in determining bony resections/cuts, implant type, component placement, and size selection [[4], [5], [6], [7], [8], [9]].

The significant anatomical alterations that occur in the later sequelae of developmental hip dysplasia, especially in high-riding dislocations—such as hypoplastic-dysplastic acetabulum, alterations in proximal femoral anatomy, leg length discrepancy, and serious soft tissue contractions—necessitate a different approach to hip arthroplasty procedures compared to standard applications [1,[10], [11], [12]]. Preoperative templating could be a valid tool for addressing critical considerations, including selecting the optimal femoral implant type and size, which must be tailored to the diverse anatomical variations of the femur. Additionally, preoperative templating, together with comprehensive preoperative planning and assessment of lower extremity parameters, provides valuable information regarding the optimal size and positioning of the acetabular component and the amount of femoral shortening required to restore limb length and achieve a stable hip [[13], [14], [15]].

Although there is a certain body of literature on manual, digital two dimensional (2D), and three dimensional (3D) templating in THA and total knee arthroplasty, there is a paucity of studies reporting the outcomes of preoperative digital templating in Crowe type 4 hips, where preoperative planning holds critical value [[16], [17], [18], [19]]. The aim of this study was to evaluate the accuracy and clinical role of digital templating applied prior to THA in Crowe type 4 hips. Specifically, the study analyzed the accuracy of templating performed by both a senior resident and a senior surgeon through data on the size of the implants used and the length of femoral shortening performed.

## Material and methods

The study was conducted in the arthroplasty unit of an orthopaedic specialty hospital. Patient data were obtained from electronic patient records covering the period from January 1, 2016, to December 31, 2019. Ethical approval was obtained from the institutional local ethics committee under code 48865165-302.14.01. The inclusion criteria included patients with a Crowe type 4 hip dysplasia, having a complete set of preoperative and postoperative pelvic radiographs, and hip arthroplasty performed with a combination of the Zimmer Wagner Cone Prosthesis Stem and Zimmer Trilogy IT Acetabular Cup (Zimmer Biomet, Warsaw, IN, USA). The exclusion criteria included cases where the femoral head was used as an autograft to create the acetabular roof, cases with a history of surgery on the same hip, cases where the acetabular component was not placed in the true acetabulum, and cases with incomplete radiological data.

Demographic data, including laterality, gender, and age, were noted. Intraoperative variables, as documented in the operation notes, including the amount of femoral shortening and the size of the implants used, were noted.

Radiological measurements were independently performed by 1 senior resident and 1 senior arthroplasty surgeon. Pelvic measurements and templating were conducted using the anteroposterior pelvic radiograph. Radiographs were obtained using a Shimadzu type R-300 collimator (Shimadzu Corporation, Kyoto, Japan) with a standard distance of 115 cm between the cassette and the tube. The Ortho View Enterprise Software (Version 6.1.2.2 IND; Meridian Technique Ltd., Southampton, UK) was utilized for measurements and digital templating. The digital radiographic magnification factor was determined using postoperative radiographs from 25 patients included in the study cohort. Measured component dimensions were compared with the known true dimensions of the implants, yielding a magnification factor of 116.8%, which was subsequently applied to all radiographic measurements.

The evaluation of the difference in vertical position of the proximal femur was based on the pelvic axis, using the teardrop as the reference point (Fig. 1). The vertical position of the proximal femurs was assessed by measuring the perpendicular distance from the inter-teardrop line (pelvic reference line) to the most prominent point of each lesser trochanter. The side-to-side difference was calculated by measurement of one hip from that of the contralateral hip. The calculation of femoral shortening was also performed on anteroposterior pelvic radiographs. Initially, the acetabular component was templated in its true position, followed by templating the femoral component without considering the amount of shortening. Subsequently, the vertical distance between the center of rotation of the templated femoral component head and the center of rotation of the templated acetabular component was measured. The amount of shortening required was determined as follows: based on the surgical planning assumption that up to 35 mm of limb lengthening may be acceptable in selected patients, the resection length for shortening was calculated by subtracting 35 mm from the measured vertical distance between the templated femoral head and the center of rotation of the templated acetabular component (Fig. 2).

**Figure 1:**
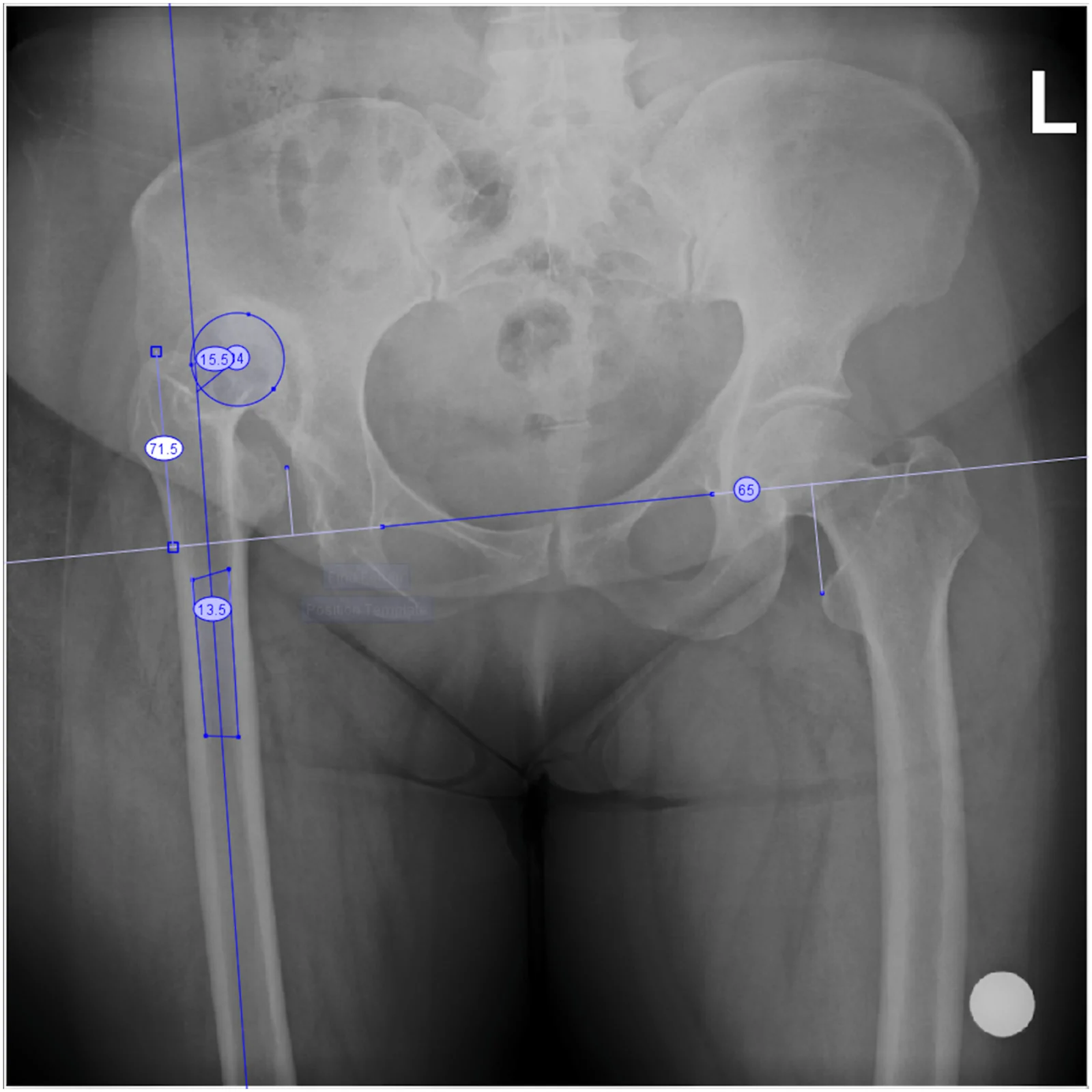
Determining the difference in vertical position of the proximal femur using teardrop and minor trochanters as references. The vertical position of the proximal femurs was assessed by measuring the perpendicular distance from the inter-teardrop line (pelvic reference line) to the most prominent point of each lesser trochanter. The side-to-side difference was calculated by measurement of one hip from that of the contralateral hip.

**Figure 2:**
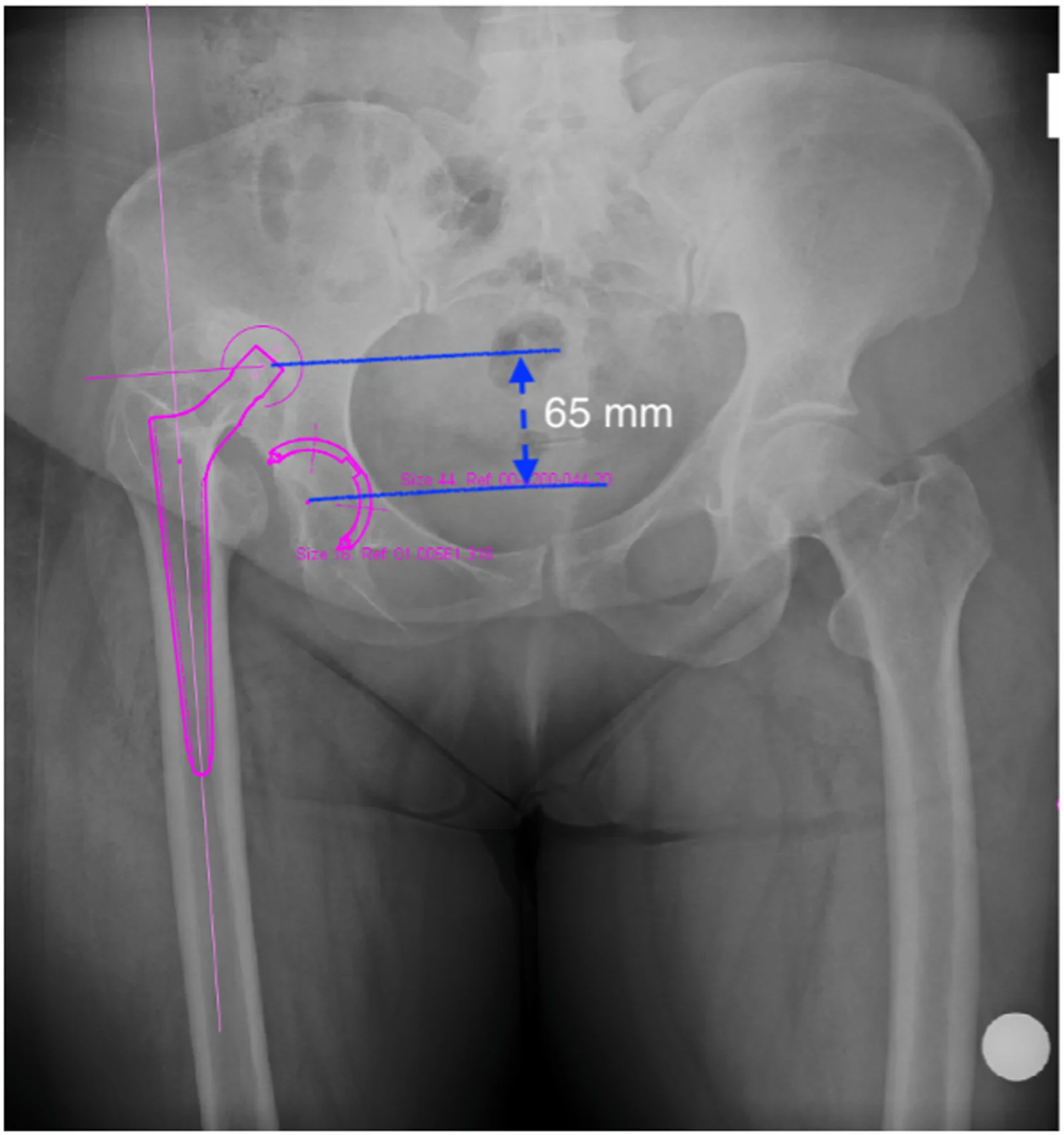
Femoral shortening was determined on anteroposterior pelvic radiographs. After templating the acetabular component in its anatomic position and the femoral component without shortening, the vertical distance between the centers of rotation of the templated femoral head and acetabular component was measured. The amount of shortening required was determined as follows: based on the surgical planning assumption that up to 35 mm of limb lengthening may be acceptable in selected patients, the resection length for shortening was calculated by subtracting 35 mm from the measured vertical distance between the templated femoral head and the center of rotation of the templated acetabular component. In this case, the required femoral shortening was calculated as 30 mm by subtracting the acceptable limb lengthening of 35 mm from the measured vertical distance of 65 mm between the templated centers of rotation.

The templating of the acetabular component was performed on the anteroposterior pelvic radiograph, with the plan to position the acetabular component in its true anatomical location. The aim was to medially rest the component on the Kohler line, using the teardrop as a reference point [20]. The target inclination angle was set at 45 degrees, and the component size was chosen to require the minimal amount of reaming necessary (at least 70%) to achieve adequate coverage for the acetabular cup. Similarly, the femoral component templating was performed on the anteroposterior pelvic radiograph. For all patients, the osteotomy configuration was planned as a subtrochanteric transverse osteotomy, 1 cm distal to the lesser trochanter. Using the software, the proximal femur was superimposed on the distal image in a manner that maintained alignment with the medullary geometry for the intended shortening (Fig. 3). The size of the femoral component was selected to achieve optimal endosteal contact within the medullary canal distal to the osteotomy, anticipating that the component would mostly fit in the narrower distal part. Care was taken to ensure that the calculated values for lengthening were not disrupted with vertical and lateral offsets.

**Figure 3:**
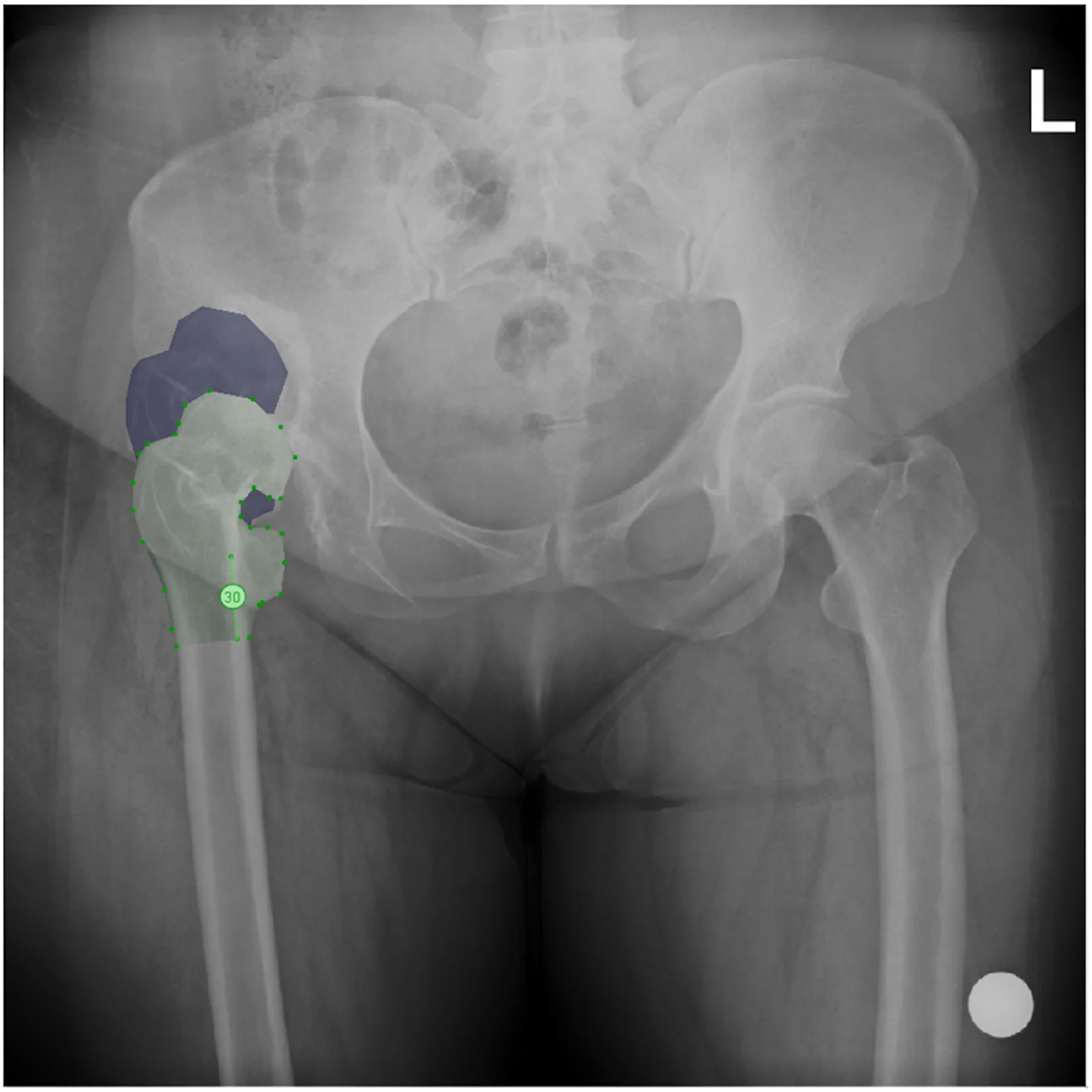
Case example of an osteotomy configuration. Osteotomy was planned as a subtrochanteric transverse osteotomy, 1 cm distal to the lesser trochanter. Using the software, the proximal femur was superimposed on the distal image in a manner that maintained alignment with the medullary geometry for the intended shortening.

To assess the accuracy of preoperative templating, several complementary analyses were performed. Digital templating was routinely performed as part of the standard preoperative planning for all patients. For the purposes of the present study, the templating measurements were reassessed retrospectively by the senior arthroplasty surgeon and the senior resident using the preoperative radiographs only. Both observers were blinded to the operative reports, postoperative radiographs, definitive implant sizes, and the actual amount of femoral shortening to minimize observer bias. For each observer, the agreement between the templated and actual femoral stem size, acetabular cup size, and planned femoral shortening was evaluated. In addition, templating accuracy was quantified by calculating the proportions of exact matches and of predictions within ±1 implant size for both the femoral stem and acetabular cup. Finally, interobserver agreement between the senior resident and the senior arthroplasty surgeon was assessed.

### Surgical technique

Patients were positioned in the lateral decubitus position, and a standard posterolateral approach was used. The short external rotators were detached, followed by femoral neck osteotomy. The iliopsoas tendon was released, and the proximal linea aspera was exposed to facilitate femoral mobilization. A subtrochanteric femoral shortening osteotomy was then performed, and the resected bone segment was preserved. Following complete capsulectomy, the true acetabulum was identified, sequentially reamed, and the acetabular component was implanted. Femoral anteversion was adjusted before insertion of the femoral stem. The final implant sizes were determined intraoperatively based on sequential acetabular reaming and femoral broaching to achieve optimal press-fit fixation and implant stability while considering the patient's anatomy and bone quality. Preoperative templating served as a guide for implant selection but did not supersede intraoperative assessment. The resected bone segment was split longitudinally and secured around the osteotomy site with cerclage cables. In selected cases, supplemental plate-and-screw fixation was used to enhance osteotomy stability (Fig. 4).

**Figure 4:**
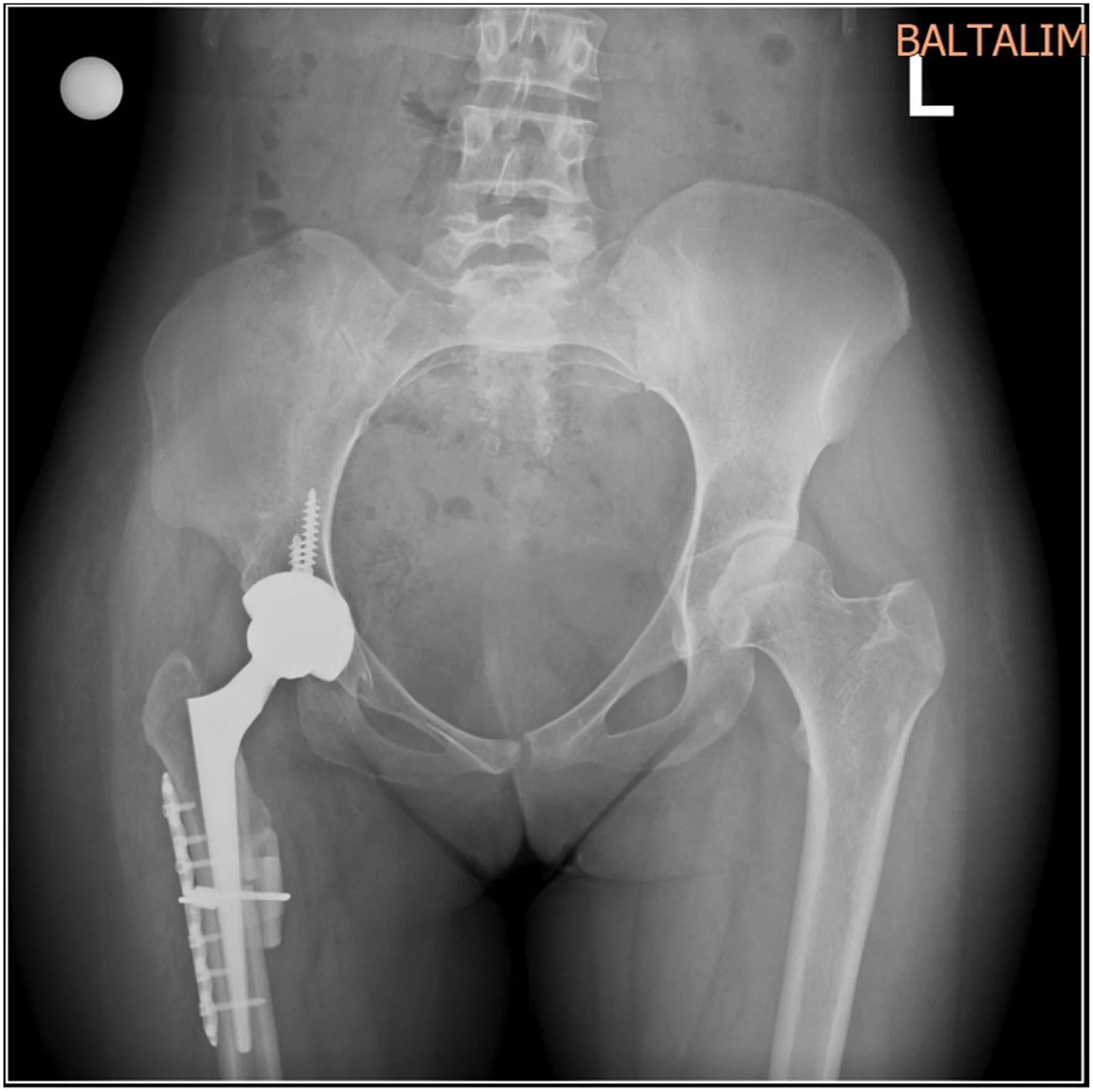
Case example of an osteotomy fixed with a cerclage and plate-screw combination.

### Statistical analysis

All statistical analyses were performed using MedCalc (version 22; MedCalc Software Ltd., Ostend, Belgium). Categorical variables are presented as frequencies and percentages, whereas continuous variables are reported as mean ± standard deviation or median with interquartile range, according to data distribution. Agreement between the templated and implanted acetabular and femoral component sizes was assessed using linear weighted Cohen's kappa (κ), as implant sizes represent ordinal categorical variables. Exact agreement was defined as the templated and implanted components being of identical size, whereas a ±1-size agreement was defined as the templated size differing by no more than one adjacent implant size from the implanted component. Kappa coefficients are presented with 95% confidence intervals (CIs), and agreement was interpreted according to the criteria of Landis and Koch (κ < 0, poor; 0.00-0.20, slight; 0.21-0.40, fair; 0.41-0.60, moderate; 0.61-0.80, substantial; 0.81-1.00, almost perfect). Statistical significance was inferred when the 95% CI did not include zero. The agreement between the templated and actual femoral shortening lengths was evaluated using the intraclass correlation coefficient (ICC) based on a two-way mixed-effects model for absolute agreement with single measurements [ICC(3,1)]. Differences between templated and actual femoral shortening lengths were assessed using the Wilcoxon signed-rank test, and the magnitude of the paired difference was reported as the Hodges–Lehmann median difference with its corresponding 95% CI. A two-sided *P* value < .05 was considered statistically significant for all analyses. No formal priori sample size calculation was performed, as this retrospective study included all eligible patients treated during the predefined study period.

## Results

A total of 50 primary total hip arthroplasties were performed in 47 patients included in the final analysis, including 3 patients who underwent bilateral procedures. The mean age at the time of surgery was 46.0 ± 9.5 years (median, 46 years; range, 21-69 years). The study cohort comprised 45 female (95.7%) and 2 male (4.3%) patients. Of the 50 hips analyzed, 29 (58.0%) were right-sided and 21 (42.0%) were left-sided (Table 1).

**Table 1:** Demographic characteristics of patients.

Variable	Value
Patients (n)	47
Hips (n)	50
Age (y), mean ± SD	45.98 ± 9.49
Age (y), median (range)	46 (21-69)
Sex, n (%)	
Female	45 (96%)
Male	2 (4%)
Side, n (%)	
Right	29 (58%)
Left	21 (42%)

SD, standard deviation.

Age and sex are reported per patient (n = 47); side is reported per hip (n = 50), as some patients underwent bilateral surgery.

Upon examining the measurements taken by the senior arthroplasty surgeon, the correlation between the templated and actual sizes of the acetabular component revealed a one-to-one match in 74% of cases. When allowing for a tolerance of ±1 size, the accuracy increased to 88%. Ten percent of the acetabular components were templated at a size larger than the implanted size, while 16% were templated at a size smaller than the actual applied size (Table 2). The senior surgeon's accuracy in acetabular component templating demonstrated a moderate level of correlation (κ = 0.410; 95% CI, 0.207-0.613). When examining the correlation between the acetabular templated sizes determined by the senior resident and the actual implanted sizes, a one-to-one agreement was observed in 44% of cases (Table 3). When allowing for a ±1 size tolerance, this agreement increased only to 62%. Additionally, 10% of the acetabular components were templated at a size larger than the actual implanted size, while 36% were templated at a size smaller. The correlation between the senior resident's templating and the actual implant size was found to be significantly poor for the acetabular component (κ = 0.071; 95% CI, −0.081 to 0.223). The linear weighted Cohen's kappa for interobserver agreement for acetabular component size was 0.191 (95% CI, 0.008-0.374), with an exact agreement rate of 52.0% (Table 4).

**Table 2:** Distribution of templated and implanted acetabular component size (mm): template by senior surgeon.

Templated/implanted	40	42	44	48	Total
40	1	1	0	0	2
42	2	0	1	0	3
44	2	0	36	3	41
46	0	0	0	3	3
48	0	0	1	0	1
Total	5	1	38	6	50

**Table 3:** Distribution of templated and implanted acetabular component Size (mm): template by senior resident.

Templated/implanted	40	42	44	48	Total
40	2	0	10	3	15
42	1	1	7	0	9
44	2	0	19	2	23
46	0	0	0	1	1
48	0	0	2	0	2
Total	5	1	38	6	50

**Table 4:** Agreement summary for acetabular component size.

Comparison	Exact match, n (%)	1-category difference, n (%)	2-category difference, n (%)	>2-category difference, n (%)	Weighted κ	SE	95% CI
Implanted–senior resident	22 (44)	9 (18)	16 (32)	3 (6)	0.071	0.078	−0.081 to 0.223
Implanted–senior surgeon	37 (74)	7 (14)	6 (12)	0 (0)	0.410	0.104	0.207-0.613
Senior resident – senior surgeon	26 (52)	10 (20)	12 (24)	2 (4)	0.191	0.093	0.008-0.374

SE, standart error.

κ = linear weighted Cohen's kappa. Category difference reflects the ordinal rank distance between templated and actual size categories (not the absolute millimeter difference). Percentages in each row sum to 100%. Kappa values were interpreted according to Landis and Koch (1977) criteria.

In the analysis of the femoral component templating data by the senior arthroplasty surgeon, the one-to-one match between the templated and actual implanted sizes was observed in only 36% of cases. However, when a tolerance of ±1 size was allowed, the compatibility rate increased to 74%. In only 1 case (2%), the templated size of the femoral component was measured larger than the actual implanted size. In contrast, in 62% of the cases, the templated size was measured smaller than the actual implanted size (Table 5). For femoral component size, the senior surgeon's preoperative template demonstrated moderate agreement with the implanted component size (linear weighted κ = 0.432; 95% CI, 0.274-0.590). In the analysis of the femoral component templating data by the senior resident, the one-to-one match between the templated and actual implanted sizes was observed in only 18% of cases. However, when a tolerance of ±1 size was allowed, the compatibility rate increased to 50%. In 4 cases (8%), the templated size of the femoral component was measured larger than the factual implanted size. In contrast, in 42% of the cases, the templated size was measured smaller than the actual implanted size (Table 6). Agreement between the templated and implanted femoral component size was slight for the senior resident (linear weighted κ = 0.265; 95% CI, 0.122-0.408). Interobserver agreement was substantial (linear weighted κ = 0.636; 95% CI, 0.497-0.776), with an exact agreement rate of 50.0% (Table 7).

**Table 5:** Distribution of templated and implanted femoral component size: template by senior surgeon.

Implanted/templated	13	14	15	16	17	18	19	Total
13	3	0	0	0	0	0	0	3
14	2	4	0	0	0	0	0	6
15	1	3	3	1	0	0	0	8
16	0	3	7	5	0	0	0	15
17	2	3	2	2	1	0	0	10
18	0	0	1	1	3	1	0	6
19	0	0	0	0	0	1	1	2
Total	8	13	13	9	4	2	1	50

**Table 6:** Distribution of templated and implanted femoral component size: template by senior resident.

Implanted∖templated	13	14	15	16	17	18	19	20	Total
13	3	0	0	0	0	0	0	0	3
14	3	1	2	0	0	0	0	0	6
15	4	2	1	1	0	0	0	0	8
16	2	7	4	2	0	0	0	0	15
17	2	4	2	1	1	0	0	0	10
18	0	0	1	3	1	1	0	0	6
19	0	0	0	0	0	1	0	1	2
20	0	0	0	0	0	0	0	0	0
Total	14	14	10	7	2	2	0	1	50

**Table 7:** Agreement summary for femoral component size.

Comparison	Exact match, n (%)	1-category difference, n (%)	2-category difference, n (%)	>2-category difference, n (%)	Weighted κ	SE	95% CI
Implanted – senior resident	9 (18)	16 (32)	16 (32)	9 (18)	0.265	0.073	0.122-0.408
Implanted – senior surgeon	18 (36)	19 (38)	7 (14)	6 (12)	0.432	0.081	0.274-0.590
Senior resident – senior surgeon	25 (50)	21 (42)	3 (6)	1 (2)	0.636	0.071	0.497-0.776

SE, standart error.

κ = linear weighted Cohen's kappa. Category difference reflects the ordinal rank distance between templated and actual size categories (not the absolute millimeter difference). Percentages in each row sum to 100%. Kappa values were interpreted according to Landis and Koch (1977) criteria.

When analyzing the variable of femoral shortening, there was a moderate level of agreement between the amount of shortening templated by the senior arthroplasty surgeon and the actual shortening applied (ICC = 0.544 [95% CI, 0.319-0.712]). In contrast, this agreement was fair for the resident's measurements (ICC = 0,400 [95% CI, 0.141-0.608]). Agreement between the 2 observers was high, with an ICC of 0.958 (95% CI, 0.928-0.976). No systematic difference was observed between measurements, with a Hodges–Lehmann median difference of 0.00 mm (95% CI, −1.00 to 1.00; *P* = .909) (Table 8).

**Table 8:** Comparison of templated and implanted femoral shortening (mm).

Comparison	Templated median [IQR]	Actual median [IQR]	HL median difference (95% CI)	p	ICC (95% CI)
Senior resident – *implanted*	30 [20-36]	30 [30-35]	1.50 (−1.50 to 4.50)	.373	0.400 (0.141-0.608)
Senior surgeon – *implanted*	30 [22-35]	30 [30-35]	1.00 (−1.00 to 3.50)	.240	0.544 (0.319-0.712)
Senior resident – senior surgeon	30 [20-36]	30 [22-35]	0.00 (−1.00 to 1.00)	.909	0.958 (0.928-0.976)

HL, Hodges-Lehmann. Differences were assessed using the Wilcoxon signed-rank test, as the data were not normally distributed. (ICC_3,1_; two-way mixed-effects, absolute agreement, single measures).

## Discussion

The most striking finding of the study is the limited accuracy of 2D digital templating in THA procedures for dysplastic Crowe type 4 hips, where patient-specific and intraoperative variables play a crucial role in surgical decision-making. Nevertheless, it has been demonstrated that preoperative 2D digital templating can offer some insights, particularly in determining the amount of femoral shortening and planning the acetabular and femoral component, when performed by an experienced surgeon.

To achieve optimal results in Crowe type 4 dysplastic hips, it is crucial to correct leg length discrepancy and select the appropriate type of femoral implant. The choice of the femoral component and its positioning are critical factors in restoring leg length, ensuring implant survival, reducing the risk of periprosthetic fracture, and enhancing overall implant survival [1,21,22]. The inherent changes in dysplastic hips, such as a narrow medullary canal and asymmetrical anatomy, necessitate careful consideration of these variables during the preoperative period, which challenges the development of a standardized and comprehensive approach. Although the predictive value of templating for femoral component size selection remains relatively low, the high correlation between the templated and applied shortening, which is also associated with femoral component positioning, highlights the importance of preoperative 2D templating in this aspect of surgical planning.

In Crowe type 4 dysplastic hips, unlike primary coxarthrosis cases, the hip rotation center is shaped by the acetabular component placed in the newly created acetabular roof, which is positioned according to more subjective parameters, guided by subtle anatomical landmarks. The restoration of femoral offset, in conjunction with the proper placement of the acetabular component, is essential for determining the biomechanics and functionality of the newly reconstructed hip joint. During the preoperative templating phase, the spatial positioning of the acetabular component, the amount of bony coverage in the planned position, and the extent of acetabular reaming required for optimal placement can be effectively simulated. We believe that the low correlation observed for the acetabular component size in our series may have been influenced by the intraoperatively assessed component fitting. Nonetheless, the high level of agreement observed when accepting a ±1 size tolerance indicates that templating the acetabular component is valuable for accurately determining the appropriate size.

Our findings are consistent with previous reports on templating in dysplastic hips; despite its limitations, conventional 2D templating remains a valuable preoperative planning tool in developmental dysplasia of the hip. Kristoffersson et al. [23] reported that digital templating was reliable for cementless THA in patients with developmental dysplasia of the hip, although their study included a broader spectrum of dysplasia rather than exclusively Crowe type 4 hips. Similarly, Zhao et al. [16] concluded that digital templating is a practical planning tool for Crowe type 2 and 3 dysplastic hips despite reduced cup-size prediction accuracy. Chen et al. [24] demonstrated that 3-dimensional simulation more accurately predicted acetabular cup size than conventional 2D templating in severely dysplastic hips and recommended its use, particularly for Crowe type 4 patients. Likewise, Lakstein et al. [14] emphasized that although meticulous preoperative planning is essential in dysplastic hips, the 3-dimensional complexity of the deformity may be underestimated on 2-dimensional radiographs. Our findings support these observations, particularly regarding the relatively low accuracy of femoral stem size prediction. Nevertheless, we demonstrated that conventional 2D templating remains valuable for planning the amount of femoral shortening in Crowe type 4 hips, with moderate agreement between the experienced surgeon's planned and actual osteotomy length. 3D imaging has become an increasingly valuable adjunct for preoperative planning in complex hip reconstruction by improving the understanding of patient-specific anatomy and facilitating implant positioning [8,[25], [26], [27], [28], [29]]. However, in our practice, 3D planning complements rather than replaces conventional 2D templating, as we continue to rely on 2D templating for implant size estimation and planning of femoral shortening. With the growing integration of artificial intelligence and robotic-assisted arthroplasty, preoperative planning is likely to evolve toward three-dimensional modeling. In this context, accurate 2D templating data will continue to play an important role in training machine learning algorithms and refining emerging technologies. Accordingly, we consider 2D and 3D planning to be complementary rather than competing modalities, and we believe that the present findings provide valuable evidence regarding the performance of conventional templating in this challenging patient population.

The correlation between the accuracy of hip arthroplasty templating and the surgeon's experience has been demonstrated in various studies on total hip arthroplasties performed for primary coxarthrosis [7]. Although different series report varying rates, it has been shown that, when a size tolerance of ±1 is accepted, experienced surgeons can predict both the acetabular and femoral component sizes with approximately 90% accuracy [9,19,29]. In our series, however, these accuracy rates were notably lower, even in templating performed by experienced surgeons, compared to templating for primary hip arthroplasties as previously reported. This discrepancy can be attributed to the inherent challenges associated with Crowe type 4 hips. Nevertheless, the influence of surgical experience on templating accuracy appears to remain relevant for total hip arthroplasties in Crowe type 4 hips.

This study has several limitations. First, its retrospective, single-center design may have introduced selection and information bias, thereby limiting the generalizability of the findings. Although consecutive eligible patients were included to minimize selection bias and standardized radiographic protocols were used for all measurements, radiographic assessment remains susceptible to errors related to patient positioning, image acquisition, and magnification. Accordingly, the possibility of measurement bias cannot be completely excluded and should be considered when interpreting the reported measurements. Second, all patients were treated using a single implant system; therefore, the findings may not be directly generalizable to other implant designs. Third, intraobserver reliability was not assessed. In addition, we did not evaluate the accuracy of other templating parameters, such as femoral implant positioning or acetabular component coverage, nor did we correlate templating accuracy with postoperative clinical outcomes. Despite these limitations, our findings provide clinically relevant information regarding the role of conventional 2D digital templating in the preoperative planning of THA for Crowe type 4 dysplastic hips.

## Conclusions

The accuracy of 2D digital templating for predicting component size in THA for Crowe type 4 dysplastic hips was limited. Nevertheless, when performed by an experienced surgeon, 2D digital templating remains a valuable preoperative planning tool for planning and estimating the required femoral shortening, while final implant selection should be based on intraoperative findings.

## Ethics approval and consent to participate

Ethical approval was obtained from local ethics committee of Baltalimanı Hospital under code “48865165-302.14.01.” Consent for participation was obtained from patients.

## Consent for publication

Consent for publication was obtained from patients.

## Availability of data and materials

Dataset for that study is available from corresponding author upon reasonable request.

## Conflicts of interest

Arman Vahabi is an editorial board member of *Journal of Orthopaedic Surgery and Research*, Minerva Orthopaedics; all other authors declare no potential conflicts of interest.

For full disclosure statements refer to https://doi.org/10.1016/j.artd.2026.102182.

## CRediT authorship contribution statement

**Ahmet Danacı:** Writing – review & editing, Writing – original draft, Methodology, Investigation, Data curation, Conceptualization. **Ferdi Dırvar:** Methodology, Investigation, Data curation, Conceptualization. **Arman Vahabi:** Writing – review & editing, Writing – original draft, Methodology. **Atakan Ezici:** Methodology, Investigation. **Yakup Alpay:** Methodology, Conceptualization. **Sarper Gürsu:** Writing – review & editing, Conceptualization.

## Figures and Tables

**Conflict of Interest Statement for Dırva:**
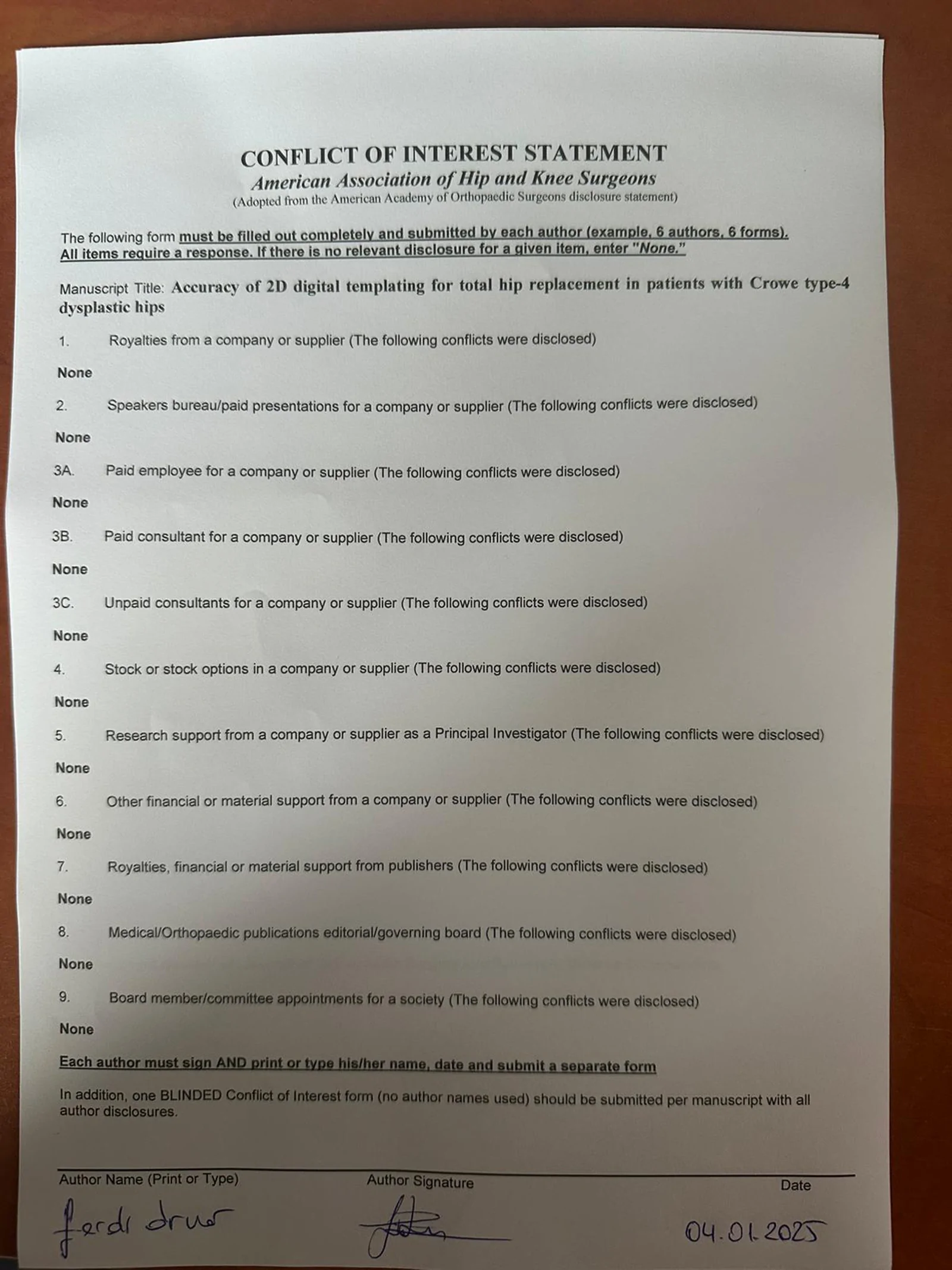


**Conflict of Interest Statement for Alpay:**
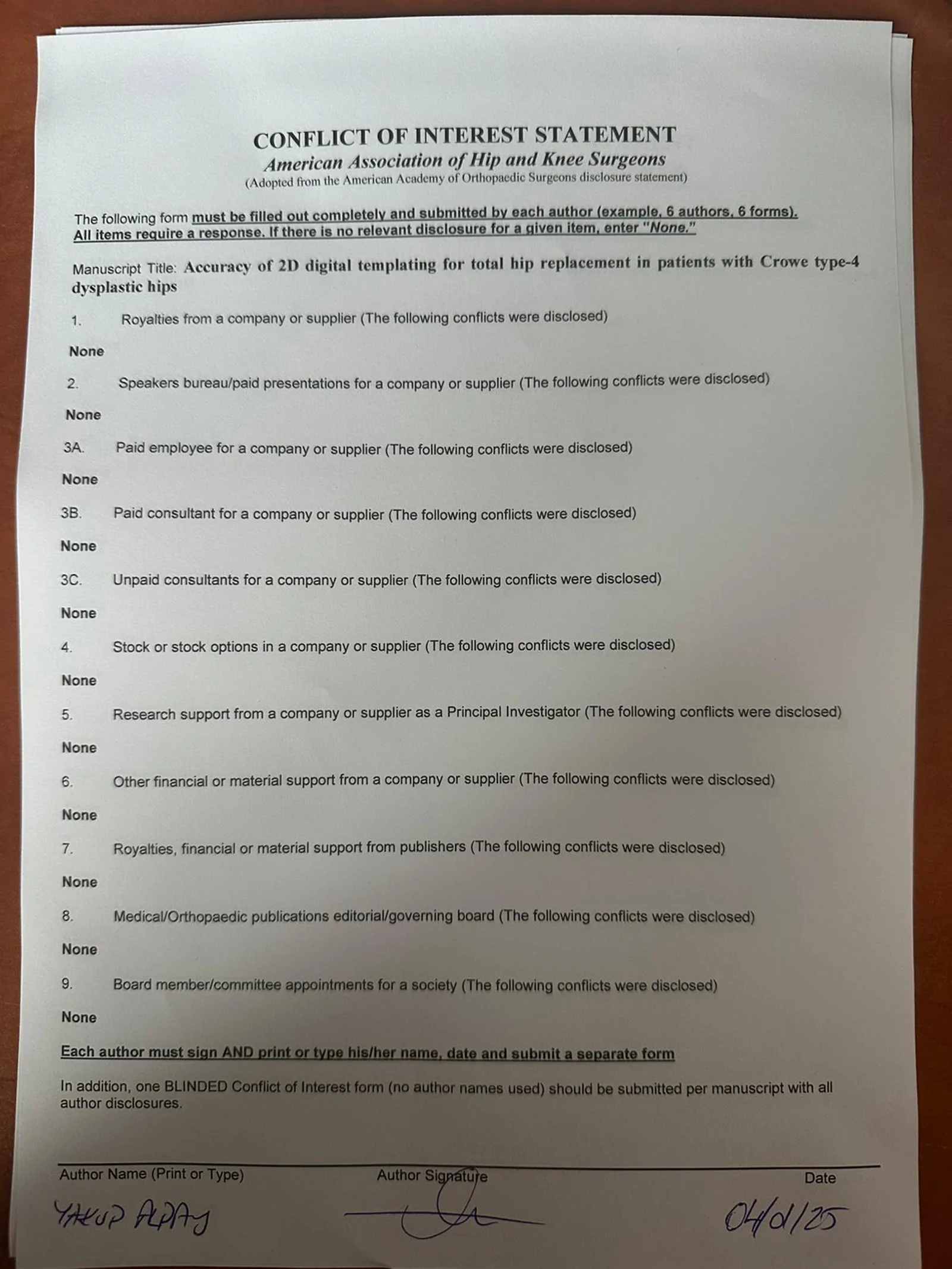


**Conflict of Interest Statement for Vahabi:**
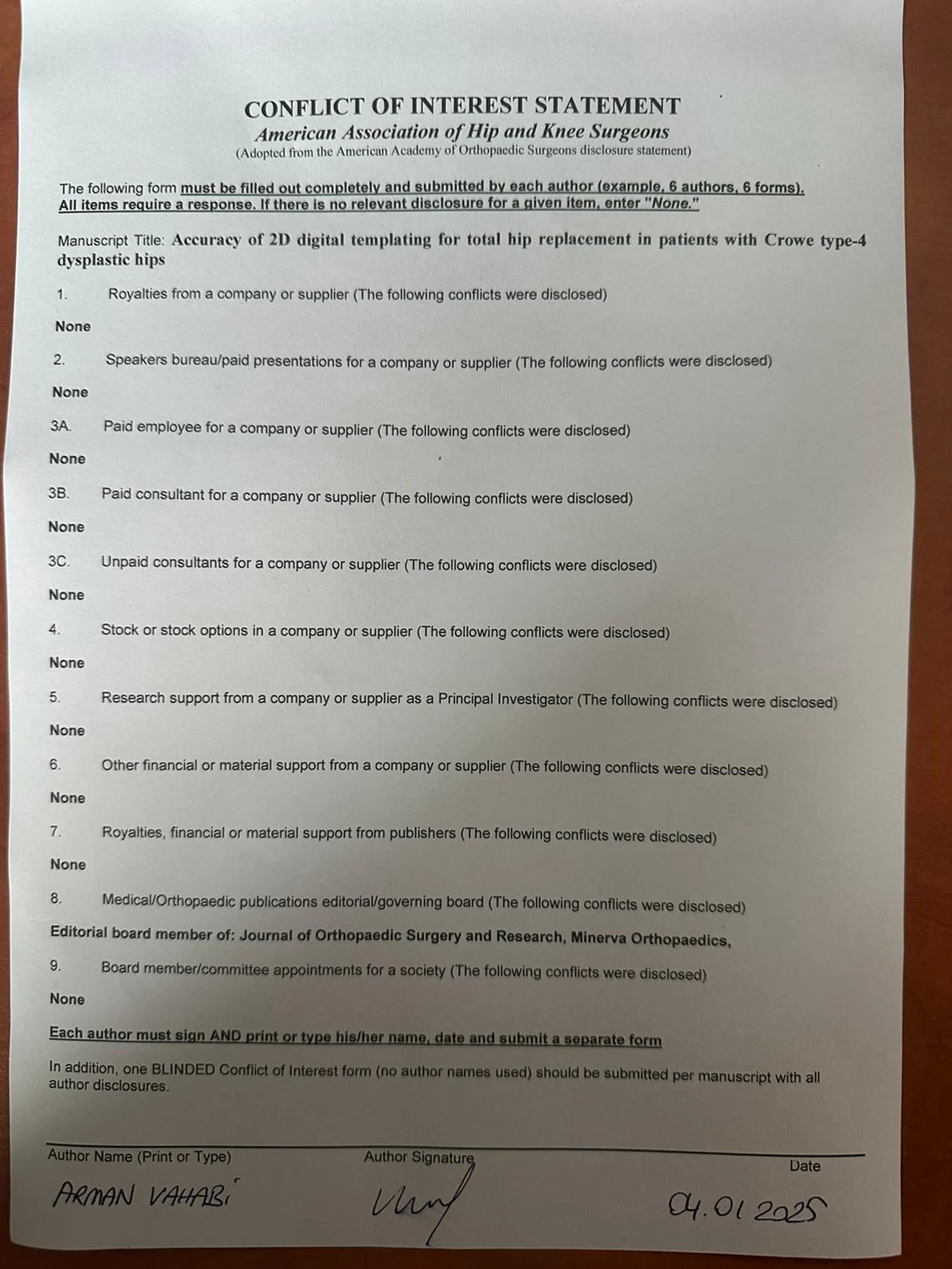


**Conflict of Interest Statement for Ezici:**
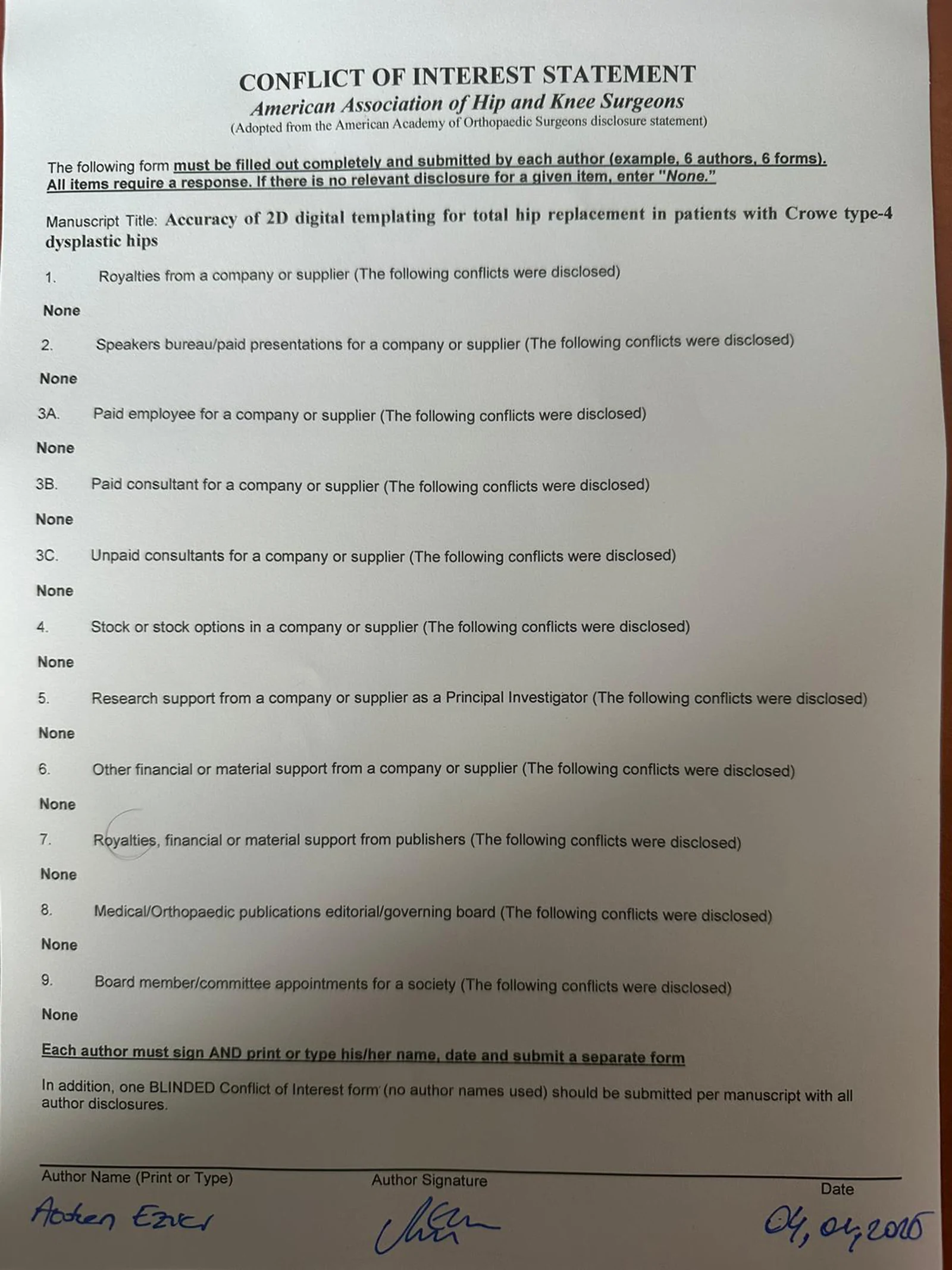


**Conflict of Interest Statement for Danacı:**
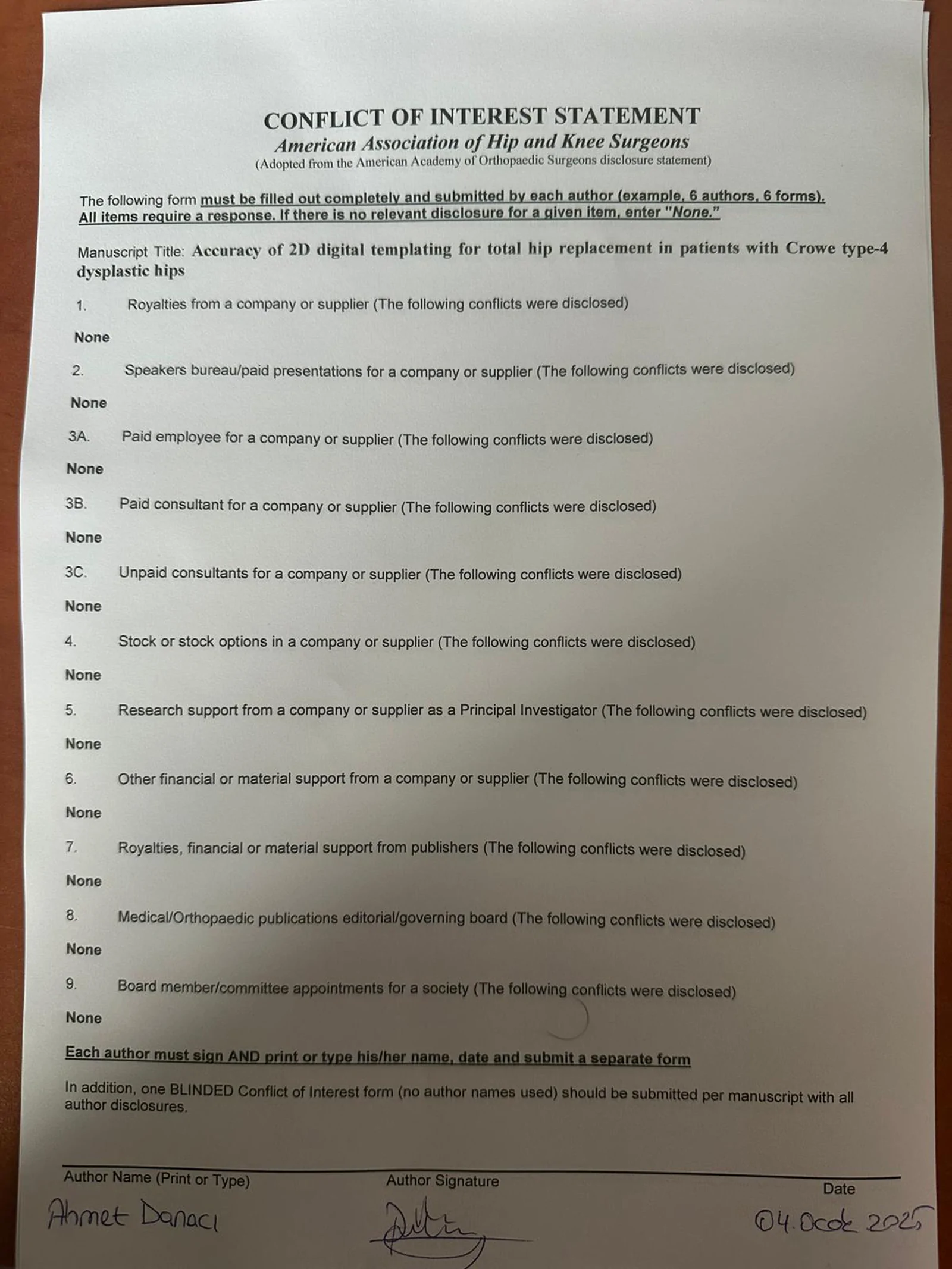


**Conflict of Interest Statement for Gürsu:**